# Elevated Levels of Oxidative Nucleic Acid Modification Markers in Urine From Gastric Cancer Patients: Quantitative Analysis by Ultra Performance Liquid Chromatography-Tandem Mass Spectrometry

**DOI:** 10.3389/fchem.2020.606495

**Published:** 2020-12-17

**Authors:** Qin Chen, Yiqiu Hu, Zhihao Fang, Minfeng Ye, Jingqing Li, Suzhan Zhang, Ying Yuan, Cheng Guo

**Affiliations:** ^1^Cancer Institute (Key Laboratory of Cancer Prevention and Intervention, China National Ministry of Education), The Second Affiliated Hospital, Zhejiang University School of Medicine, Hangzhou, China; ^2^Department of Gastrointestinal Surgery, Shaoxing People's Hospital, Shaoxing Hospital of Zhejiang University, Shaoxing, China; ^3^Zhejiang University-University of Edinburgh Institute, Zhejiang University School of Medicine, Haining, China; ^4^Department of Medical Oncology, The Second Affiliated Hospital, Zhejiang University School of Medicine, Hangzhou, China

**Keywords:** UPLC-MS/MS, nucleic acid modification, oxidative stress, gastric cancer, biomarker, urine

## Abstract

Oxidative nucleic acid modifications have attracted increasing attention in recent years since they have been found to be related to a number of diseases including cancer. 8-Hydroxy-2′-deoxyguanosine (8-OHdG) and 8-hydroxyguanosine (8-OHG) are the typical markers of oxidative modification of DNA and RNA, respectively, and they are emerging biomarkers for the early detection of diseases. Urine is a favored biofluid for biomarker discovery due to its noninvasiveness to patients. Accurate quantification of these oxidative nucleic acid modifications still has challenges because their amounts in urine are very low and the interferences in urine samples are complicated. Herein, we developed and validated an accurate and robust solid-phase extraction (SPE) coupled with ultra performance liquid chromatography-tandem mass spectrometry (UPLC-MS/MS) method for the simultaneous quantification of these oxidative nucleic acid modifications in human urine. Stable isotope dilution strategy was utilized and the method shows good precision on intraday and interday measurements. Meanwhile, recovery was satisfactory by utilizing the Oasis hydrophilic–lipophilic balance (HLB) cartridge for sample pretreatment at three spiked levels. We successfully quantified urinary 8-OHdG and 8-OHG from 60 gastric cancer patients and 70 healthy controls by using this method. The measured contents of 8-OHdG and 8-OHG in urine from gastric cancer patients are both increased, compared with those in urine from healthy controls, indicating these oxidative nucleic acid modifications could act as potential non-invasive markers for early diagnosis of gastric cancer. Moreover, the present study will stimulate investigations of the effects of oxidative stress and nucleic acid modifications on the initiation and progression of gastric cancer.

## Introdution

Nucleic acid modifications play crucial roles in a number of biological processes and continuous efforts have been devoted to this field (Frye et al., 2018; Luo et al., 2018; Delaunay and Frye, 2019). Until now, more than 50 and 160 types of chemical modifications in DNA and RNA have been found, respectively, and these modifications could regulate the structures and functions of nucleic acids (Roundtree et al., 2017; Boccaletto et al., 2018; Sood et al., 2019). Reactive oxygen species (ROS) produced by environmental factors and cell metabolism processes can attack nucleic acids, leading to the oxidative damage of DNA and RNA (Sies and Jones, 2020). As the typical oxidative modifications of nucleic acids, 8-hydroxy-2′-deoxyguanosine (8-OHdG) and 8-hydroxyguanosine (8-OHG) have drawn increasing attention since previous studies indicated that oxidative nucleic acid damage was associated with a variety of diseases, including neurodegenerative diseases, type 2 diabetes, cardiovascular diseases, aging, and cancer (Nunomura et al., 2012; Gao et al., 2019; Liang et al., 2020; Urbaniak et al., 2020). From this point of view, 8-OHdG and 8-OHG have great potential in the early detection of diseases, apart from being indicators of endogenous oxidative nucleic acid damage.

Compared with serum and tissue samples, urine is easily obtained in large amount and the sample collection process is non-invasive to patients. Therefore, urine is a very suitable biofluid for biomarker discovery (Bulacio and Torres, 2019; Reeves et al., 2019). Due to its strong capabilities in sensitivity and selectivity, liquid chromatography-tandem mass spectrometry-based (LC-MS/MS) techniques have been widely used in the qualitative and quantitative analysis of biomolecules in urine to evaluate their potential as biomarkers in the past decades (Rodríguez-Gonzalo et al., 2016; Simpson et al., 2020). Although other analytical methods, e.g., enzyme-linked immunosorbent assay (ELISA), were utilized to analyze 8-OHdG and 8-OHG (Kobayashi et al., 2015; Sliwinska et al., 2016), the limited specificity which often leads to the overestimation of the concentrations is a major problem. As a powerful analytical tool, the stable isotope dilution LC-MS/MS technique can achieve high accuracy because the analyte loss during the pretreatment procedure and the ionization efficiency of analytes in mass spectrometer are compensated (Guo et al., 2018a). In order to improve the separation efficiency and make the analysis time shorter, substitution of conventional high-performance liquid chromatography (HPLC) by ultra-performance liquid chromatography (UPLC) is a commonly used strategy. In the past decades, UPLC coupled with tandem mass spectrometry (UPLC-MS/MS) plays vital roles in the analysis of various types of samples (Guo et al., 2018b).

Prior to UPLC-MS/MS analysis, pretreatment of urine samples is required to remove the inorganic salts that would decrease the ionization efficiency and organic interferent that would reduce the selectivity. In addition, enrichment of the analytes of interest is usually necessary to enhance LC-MS/MS detection, especially for those metabolites with low concentrations. Solid-phase extraction (SPE), as a sample pretreatment tool, is commonly utilized for sample cleaning in biomedical analysis (Andrade-Eiroa et al., 2016; Neale et al., 2018). Many types of sorbents could be used for packing the cartridges, and it was selected on the basis of the property of the target analytes.

Gastric cancer is the fifth most common cancer diagnosed globally in 2019 (Bray et al., 2018). Patients with early gastric cancer have no obvious symptoms, and thus, most patients are diagnosed when they have reached advanced stage. Early detection of gastric cancer can significantly reduce the incidence and mortality. Currently, gastroscopy is the most widely utilized approach for gastric cancer screening (Akahoshi et al., 2018; Liao et al., 2018). However, the compliance of gastroscopy is not satisfied due to its great invasion. In the current study, we developed and validated an accurate and robust SPE coupled with stable isotope dilution UPLC-MS/MS method for the simultaneous determination of urinary 8-OHdG and 8-OHG from gastric cancer patients. The levels of these two modified nucleosides in urine from 60 gastric cancer patients and 70 healthy controls were accurately measured. Moreover, we carried out statistical analysis to assess the potential of 8-OHdG and 8-OHG as non-invasive indicators for early detection of gastric cancer.

## Materials and Methods

### Chemicals and Reagents

Methanol (MeOH) of HPLC grade was obtained from Merck KGaA (Darmstadt, Germany). Formic acid (HCOOH), acetic acid (CH_3_COOH), and 8-OHdG were bought from Sigma-Aldrich (St Louis, MO, USA). [^15^N_5_]8-Hydroxy-2′-deoxyguanosine ([^15^N_5_]8-OHdG) was purchased from Cambridge Isotope Laboratories Inc. (Andover, MA, USA). 8-OHG and [^13^C^15^N_2_]8-hydroxyguanosine ([^13^C^15^N_2_]8-OHG) were bought from Toronto Research Chemical (Toronto, Canada). Water was gained from a Milli-Q water purification apparatus (Millipore, Milford, MA, USA).

### Standard Preparation

The standards of 8-OHdG, 8-OHG, [^15^N_5_]8-OHdG, and [^13^C^15^N_2_]8-OHG were dissolved in water separately at a concentration of 10 μM. Mixed working standard solutions of 8-OHdG and 8-OHG (1 μM each) were prepared by diluting standard stock solutions with water. For calibration curve establishment, the mixed working standard solution was successively diluted (1.0, 2.5, 5.0, 10.0, 25.0, 50.0, 100.0, 150.0, 200.0 nM) and then isotope-labeled internal standards were spiked (50 nM each). We also prepared quality control (QC) samples (10, 50, 200 nM) in triplicate and internal standards (50 nM each) were added.

### Clinical Sample Collection

Healthy controls were recruited from The Second Affiliated Hospital, Zhejiang University School of Medicine (SAHZU), and gastric cancer patients were recruited from Shaoxing People's Hospital, Shaoxing Hospital of Zhejiang University. Healthy volunteers did not suffer from any type of cancer. Gastric cancer patients were confirmed by a pathologist and were not treated with chemotherapy, radiotherapy, or surgery. Seventy healthy subjects (mean age of 50.0 ± 9.5 years, range 35–73 years) and 60 gastric cancer patients (mean age of 66.1 ± 8.6 years, range 51–81 years) were enlisted (Supplementary Table 1). We collected midstream urine samples in the morning and store them at −80°C. Besides, we measured the urinary creatinine levels at the Department of Laboratory Medicine, SAHZU for correction (Wu and Li, 2016).

### Urine Sample Pretreatment

The urine samples were fully thawed at room temperature, followed by centrifugation at 13,000 rpm for 15 min at 4°C. An equal volume of water was added to 200 μl of supernatant, and then [^15^N_5_]8-OHdG and [^13^C^15^N_2_]8-OHG (10 pmol each) were added. The Oasis hydrophilic–lipophilic balance (HLB) (1.0 ml, 30 mg) cartridge (Waters, Milford, MA, USA) was used for urine sample pretreatment. After being preconditioned with 1.0 ml of methanol and 1.0 ml of water successively, cartridges were loaded with urine samples. The cartridges were washed by 0.8 ml of water, and then eluted by 0.8 ml of methanol/water of 1:1 (v/v). The elution was collected and dried under vacuum and then dissolved in 200 μl of water for UPLC-MS/MS detection.

### UPLC-MS/MS Analysis

Chromatographic separation was implemented by using a Waters BEH C18 (100 mm × 2.1 mm, 1.7 μm) column, and an Acquity UPLC system (Waters, Milford, MA, USA) was utilized. We used an isocratic mode of 95% A (H_2_O containing 0.05% formic acid and 0.05% acetic acid) and 5% B (MeOH containing 0.05% formic acid and 0.05% acetic acid), and the flow rate was 0.25 ml/min. Samples were kept at 4°C and measured three times. The injection volume was 5 μl.

A 4000 QTRAP mass spectrometer (AB SCIEX, Foster City, CA, USA) was used for data acquisition, and it was ran in ESI-positive ion mode. Quantification of 8-OHdG and 8-OHG was carried out in multiple reaction monitoring (MRM) mode by monitoring the transitions of *m*/*z* 284.1 → 168.0 and *m*/*z* 300.1 → 168.0, respectively. For internal standards, the transitions of *m*/*z* 289.1 → 173.0 ([^15^N_5_]8-OHdG) and *m/z* 303.1 → 171.0 ([^13^C^15^N_2_]8-OHG) were monitored. The spray voltage was set as 5.5 kV and ion source temperature was set as 550°C. The ion source gas 1, ion source gas 2, and curtain gas were set as 60, 40, and 35 psi, respectively. The optimized MRM parameters are summarized in Supplementary Table 2.

### Method Validation

Calibration curves were constructed by plotting the peak area ratio of the analyte to the corresponding internal standard vs. the concentration of the analyte. The accuracy and intra- and interday precision of the method were assessed by analyzing the QC samples. Accuracy was determined by comparing the measured values to the theoretical concentrations. Intra- and interday precision were gained by analyzing the QC samples within 1 day and in 3 consecutive days, respectively, and expressed as the relative standard deviation. The limits of detection (LODs) and limits of quantification (LOQs) were defined as the levels of the analytes at a signal-to-noise ratio of 3 and 10, respectively. The extraction recovery was evaluated by spiking standards at three different concentrations (5, 30, 150 nM) into urine samples. After spiking with internal standards (10 pmol each), solid-phase extraction was carried out and then urine samples were analyzed. The matrix effect was assessed by constructing calibration curves in water or urine extracts, and then the slopes were compared.

### Statistical Analysis

SPSS Statistics 20.0 software (IBM, Armonk, NY, USA) was used for statistical analyses. The differences in 8-OHdG and 8-OHG concentrations between healthy controls and gastric cancer patients were assessed by Mann–Whitney *U* test, and statistical significance was considered if *p* < 0.05.

## Results and Discussion

### Optimization of the UPLC-MS/MS Method

To achieve satisfactory sample separation and sensitive mass spectrometry detection, the UPLC-MS/MS method was optimized. To gain better resolution and shorter analysis time, we used the Waters BEH C18 (100 mm × 2.1 mm, 1.7 μm) column. Organic solvents were also tested and methanol exhibited better separation performance between interferences and analytes, in comparison with acetonitrile. The mobile phase additive plays vital roles in chromatographic separation and subsequent MS detection. In previous studies, it has been demonstrated that acetic acid could improve the sensitivity of 8-OHdG in UPLC-MS/MS, whereas formic acid could improve the detection sensitivity of 8-OHG (Guo et al., 2016, 2020). Therefore, 0.05% formic acid and 0.05% acetic acid were both added into the mobile phases. Under this condition, the LOD value can reach 0.2 nM for 8-OHdG and 0.3 nM for 8-OHG. The results were lower than previously reported values (Cervinkova et al., 2017; Li et al., 2019), indicating the analytical method has excellent sensitivity.

**Figure 1:**
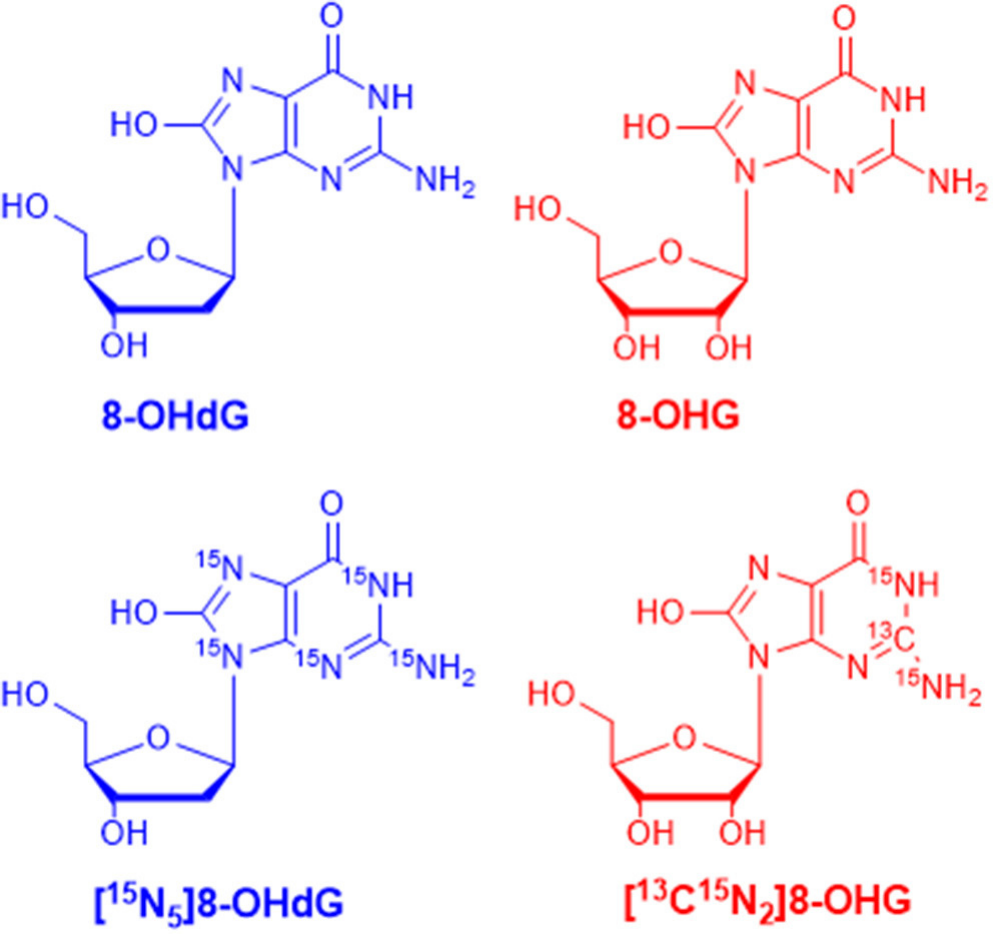
The chemical structures of 8-hydroxy-2′-deoxyguanosine (8-OHdG), 8-hydroxyguanosine (8-OHG), and the corresponding isotope-labeled internal standards.

MRM parameters were optimized by infusing the standard solution into the mass spectrometer through a peristaltic pump. In full scan ESI-MS, abundant [M+H]^+^ ions were observed. Then, collision-induced dissociation (CID) experiments were carried out. The chemical structures of 8-OHdG, 8-OHG, and the corresponding isotope-labeled internal standards are illustrated in Figure 1. The ribose group was easily eliminated from protonated molecular ions through C–N bond cleavage in MS/MS, i.e., 116 or 132 Da was lost from [M+H]^+^ ion of 8-OHdG or 8-OHG, respectively. Hence, *m*/*z* 284.1 → 168.0 was used for the quantification of 8-OHdG, and *m*/*z* 300.1 → 168.0 was monitored for the quantification of 8-OHG. Besides, *m*/*z* 289.1 → 173.0 and *m*/*z* 303.1 → 171.0 were monitored for [^15^N_5_]8-OHdG and [^13^C^15^N_2_]8-OHG, respectively. The MRM parameters including collision energy were also optimized and shown in Supplementary Table 2.

### Validation of the Analytical Method

Calibration curves were constructed in the light of previously mentioned procedures. In the range from 1.0 to 200.0 nM, excellent linearities (*R*^2^ > 0.999) were obtained. The results of the calibration curves are given in Table 1. For matrix effect, calibration curves were also constructed from urine extracts and the slopes were compared with those of calibration curves constructed in water (Supplementary Figure 1). The slope ratio values for 8-OHdG and 8-OHG were 89.0 and 88.8%, respectively. The results imply that the matrix effect on the determination of 8-OHdG and 8-OHG was negligible.

**Table 1:** Linearity of 8-OHdG and 8-OHG in the developed UPLC-MS/MS analysis method.

	**Linear equation**	***R*^**2**^ value**	**Linear range (nM)**
8-OHdG	*y* = 0.0313*x* – 0.0187	0.9998	1–200
8-OHG	*y* = 0.0237*x* + 0.0044	0.9996	1–200

As shown in Table 2, the intraday precision value was within 3.0% and interday precision value was within 2.2%. The accuracy of the intra- and interday analysis ranged from 96.0 to 106.0%. These results indicate excellent reproducibility and accuracy were achieved.

**Table 2:** Precision (intra- and interday) and accuracy of the developed UPLC-MS/MS method for the analysis of 8-OHdG and 8-OHG.

**QC**	**Theoretical values (nM)**	**Intraday (*****n*** **=** **9)**			**Interday (*****n*** **=** **3)**		
		**Mean ± SD (nM)**	**RSD (%)**	**Accuracy (%)**	**Mean ± SD (nM)**	**RSD (%)**	**Accuracy (%)**
8-OHdG	10 (low)	10.05 ± 0.30	2.94	100.49	10.07 ± 0.14	1.37	100.72
	50 (medium)	52.00 ± 0.58	1.12	103.99	51.94 ± 0.55	1.06	103.88
	200 (high)	196.32 ± 2.04	1.04	98.16	195.79 ± 4.06	2.07	97.89
8-OHG	10 (low)	9.68 ± 0.08	0.79	96.83	9.72 ± 0.06	0.58	97.17
	50 (medium)	52.87 ± 0.22	0.42	105.74	52.00 ± 1.10	2.11	103.99
	200 (high)	200.95 ± 3.07	1.53	100.47	200.79 ± 3.87	1.93	100.39

Solid-phase extraction was utilized for the sample pretreatment since urine contains inorganic salts and organic interference compounds. The HLB cartridge, which possesses superior reversed-phase capacity, was utilized. In addition, urine samples were spiked with isotope-labeled internal standards, and thus, the loss of target analytes during sample preparation was corrected. Therefore, satisfactory recovery which ranged from 87.6 to 109.8% at three spiking levels was obtained (Table 3).

**Table 3:** Recovery at three different spiking levels of the developed SPE coupled with the UPLC-MS/MS method.

	**Spiked amount (nM)**	**Mean ± SD (nM)**	**Average recovery (%)**	**RSD (%)**
8-OHdG	0	11.14 ± 0.61	–	5.48
	5 (low)	16.06 ± 0.19	98.33	1.20
	30 (medium)	43.82 ± 1.05	108.92	2.40
	150 (high)	175.81 ± 3.29	109.78	1.87
8-OHG	0	23.75 ± 1.46	–	6.14
	5 (low)	28.60 ± 1.36	96.95	4.77
	30 (medium)	52.81 ± 1.74	96.86	3.30
	150 (high)	155.17 ± 4.00	87.61	2.58

A QC sample was measured every 20 samples to monitor the system stability during measurement. Retention time and accuracy were checked and the results indicated that the system stability was excellent. In summary, the established UPLC-MS/MS method was sensitive, accurate, and robust for the simultaneous determination of 8-OHdG and 8-OHG in human urine.

### Quantification of Oxidative Nucleic Acid Modifications in Urine Samples

**Figure 2:**
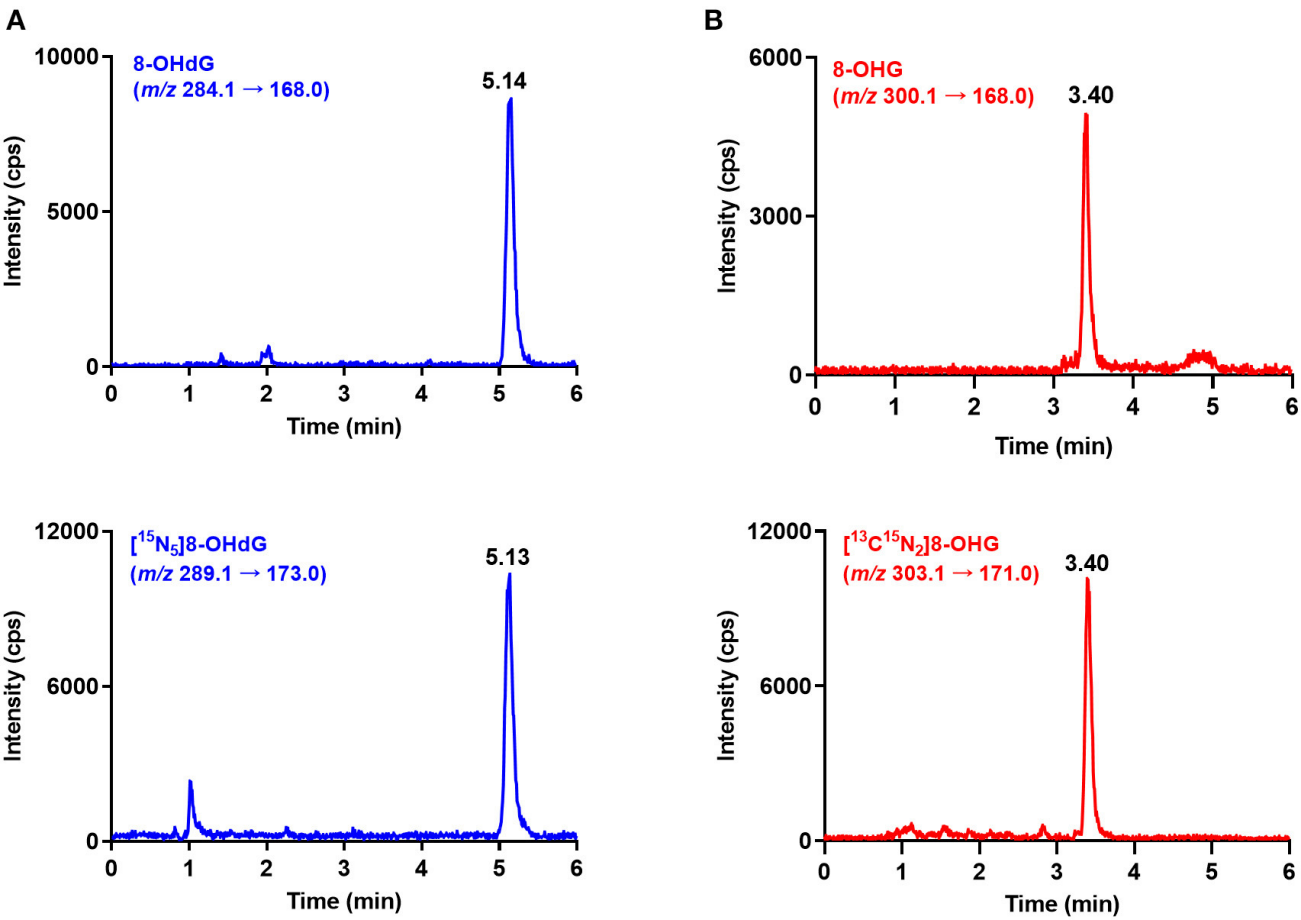
Representative MRM chromatograms of 8-OHdG **(A)**, 8-OHG **(B)** and spiked isotope-labeled internal standards in a urine sample.

Urine samples from 60 gastric cancer patients and 70 healthy controls were measured by using the developed and validated method. The retention time of 8-OHdG and 8-OHG in urine samples is identical to that of the corresponding internal standard (Figure 2), and this confirmed the existence of these oxidative nucleic acid modifications. The contents of 8-OHdG and 8-OHG were quantified according to the calibration curves and normalized against the urinary creatinine concentration. The quantification results showed that the levels of 8-OHdG and 8-OHG in human urine ranged from 0.31 to 4.98 and 0.52 to 4.86 nmol/mmol creatinine, respectively. The detailed concentrations are listed in Supplementary Table 1.

**Figure 3:**
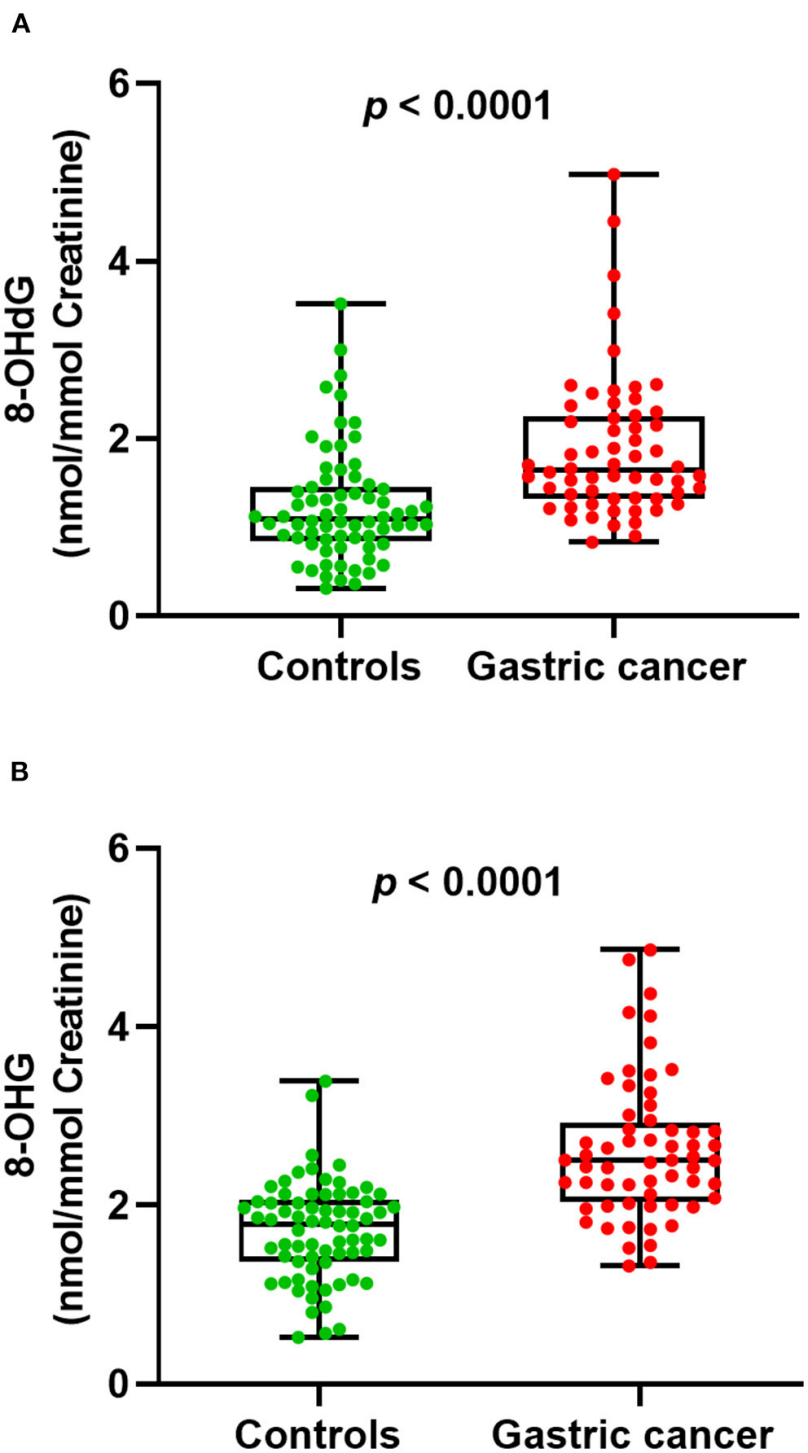
The measured concentrations of 8-OHdG **(A)** and 8-OHG **(B)** in urine samples and statistical analysis.

Next, we examined whether there were level differences of these oxidized nucleosides in gastric cancer patients and healthy volunteers. On this score, 130 urine samples from 70 healthy controls and 60 gastric cancer patients were analyzed. The measured mean concentrations of 8-OHdG and 8-OHG in urine samples from healthy controls were 1.23 ± 0.64 and 1.72 ± 0.55 nmol/mmol creatinine, respectively. For gastric cancer patients, the measured mean concentrations of 8-OHdG and 8-OHG were 1.88 ± 0.81 and 2.62 ± 0.79 nmol/mmol creatinine, respectively. The results demonstrated that compared with those in healthy controls, the concentrations of urinary 8-OHdG and 8-OHG were significantly elevated in gastric cancer patients (*p* < 0.0001, Figure 3).

**Figure 4:**
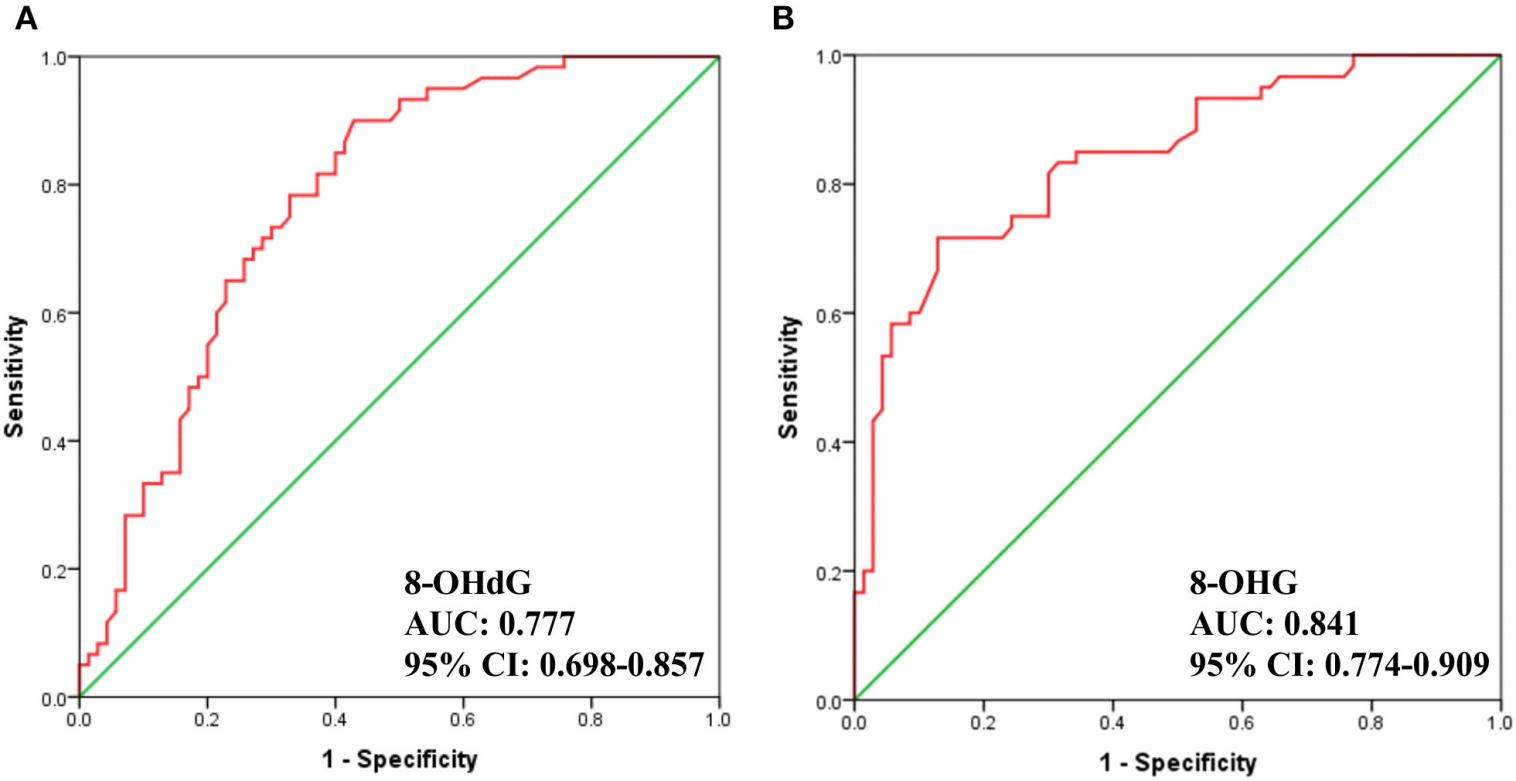
ROC analysis for 8-OHdG **(A)** and 8-OHG **(B)** in urine samples.

Additionally, we carried out receiver operator characteristic (ROC) curve analysis to evaluate the potential of 8-OHdG and 8-OHG to distinguish gastric cancer patients from healthy volunteers. As demonstrated in Figure 4, the area under the curve (AUC) is 0.777 and 0.841, respectively. The results indicated a correlation between the levels of urinary 8-OHdG and 8-OHG and the incidence of gastric cancer. These results implied that 8-OHdG and 8-OHG may act as potential non-invasive indicators for the screening of gastric cancer. In addition, oxidative nucleic acid damage plays vital roles in many physiological processes, and the increased concentrations of urinary 8-OHdG and 8-OHG from gastric cancer patients suggest that oxidative damage may be related to the occurrence and development of gastric cancer.

## Conclusions

In the present work, an accurate and robust UPLC-MS/MS method was established for the simultaneous quantification of oxidative nucleic acid modifications in urine samples from 70 healthy controls and 60 gastric cancer patients. The results showed that the contents of 8-OHdG and 8-OHG in urine from gastric cancer patients were elevated, compared with those in urine from healthy controls. The present work suggests that the increase of oxidative nucleic acid modifications in urine may be non-invasive indicators for the early detection of gastric cancer.

## Data Availability Statement

The raw data supporting the conclusions of this article will be made available by the authors, without undue reservation.

## Ethics Statement

The studies involving human participants were reviewed and approved by the Institutional Review Board of Medical Research, The Second Affiliated Hospital, Zhejiang University School of Medicine (SAHZU) and Shaoxing People's Hospital, Shaoxing Hospital of Zhejiang University. The patients/participants provided their written informed consent to participate in this study.

## Author Contributions

CG and YY designed the study. CG, QC, and YH performed the experiments. CG and QC wrote the manuscript and prepared the figures. ZF and MY collected the urine samples. JL and SZ edited the manuscript. All authors approved the submission.

## Conflict of Interest

The authors declare that the research was conducted in the absence of any commercial or financial relationships that could be construed as a potential conflict of interest.
