# Supplementary material for: Elevated Levels of Oxidative Nucleic Acid Modification Markers in Urine From Gastric Cancer Patients: Quantitative Analysis by Ultra Performance Liquid Chromatography-Tandem Mass Spectrometry
Source: Front Chem. 2020 Dec 17;8:606495. doi: 10.3389/fchem.2020.606495 (PMC7773832; doi:10.3389/fchem.2020.606495)
Supplement: Supplementary file 1 [file Data_Sheet_1.doc]

**Elevated Levels of Oxidative Nucleic Acid Modification Markers in Urine from Gastric Cancer Patients: Quantitative Analysis by Ultra Performance Liquid Chromatography-Tandem Mass Spectrometry**

Qin Chen^1#^, Yiqiu Hu^1#^, Zhihao Fang^1^, Minfeng Ye^2^, Jingqing Li^1,3^, Suzhan Zhang^1^, Ying Yuan^1,4*^, Cheng Guo^1*^

^1^Cancer Institute (Key Laboratory of Cancer Prevention and Intervention, China National Ministry of Education), The Second Affiliated Hospital, Zhejiang University School of Medicine, Hangzhou, Zhejiang 310009, China

^2^Department of Gastrointestinal Surgery, Shaoxing People’s Hospital, Shaoxing Hospital of Zhejiang University, Shaoxing, Zhejiang 312000, China

^3^Zhejiang University-University of Edinburgh Institute, Zhejiang University School of Medicine, Haining, Zhejiang 314400, China

^4^Department of Medical Oncology, The Second Affiliated Hospital, Zhejiang University School of Medicine, Hangzhou, Zhejiang 310009, China

^#^ These authors contributed equally.

*Corresponding author:

Ying Yuan, yuanying1999@zju.edu.cn;

Cheng Guo, cheng_guo@zju.edu.cn

Tel.: +86-571-87784501; Fax: +86-571-87214404.

**Supplementary Information**

**Table S1.** Levels of 8-OHdG and 8-OHG in urine from healthy controls and gastric cancer patients.

**Table S2.** The optimized MS conditions used for the analysis of 8-OHdG and 8-OHG.

**Figure S1.** Calibration curve of 8-OHdG and 8-OHG standard obtained in water and human urine for the evaluation of matrix effect.

**Table S1. Levels of 8-OHdG and 8-OHG in urine from healthy controls and gastric cancer patients.**

| **NO.** | **8-OHdG**  **(nmol/mmol creatinine)** | **8-OHG**  **(nmol/mmol creatinine)** |
| --- | --- | --- |
| N1 | 1.29±0.02 | 1.97±0.04 |
| N2 | 0.77±0.02 | 1.56±0.05 |
| N3 | 0.86±0.01 | 1.03±0.04 |
| N4 | 0.54±0.03 | 1.47±0.08 |
| N5 | 1.02±0.03 | 1.92±0.10 |
| N6 | 0.40±0.01 | 0.60±0.01 |
| N7 | 1.15±0.01 | 1.54±0.09 |
| N8 | 1.05±0.03 | 2.45±0.09 |
| N9 | 1.02±0.02 | 2.12±0.11 |
| N10 | 1.23±0.05 | 1.77±0.08 |
| N11 | 0.94±0.00 | 2.27±0.23 |
| N12 | 1.13±0.00 | 2.14±0.09 |
| N13 | 0.89±0.02 | 1.94±0.08 |
| N14 | 1.33±0.03 | 2.55±0.08 |
| N15 | 1.01±0.01 | 2.13±0.06 |
| N16 | 1.37±0.04 | 2.20±0.04 |
| N17 | 0.87±0.00 | 1.77±0.06 |
| N18 | 0.80±0.01 | 1.17±0.02 |
| N19 | 1.06±0.09 | 1.09±0.08 |
| N20 | 0.77±0.06 | 2.13±0.07 |
| N21 | 1.17±0.02 | 1.49±0.01 |
| N22 | 1.1±0.030 | 1.86±0.06 |
| N23 | 1.24±0.02 | 2.11±0.12 |
| N24 | 2.01±0.01 | 1.49±0.05 |
| N25 | 0.87±0.03 | 2.02±0.07 |
| N26 | 1.91±0.02 | 1.96±0.06 |
| N27 | 1.02±0.02 | 1.37±0.06 |
| N28 | 1.02±0.05 | 1.46±0.07 |
| N29 | 0.56±0.02 | 1.58±0.04 |
| N30 | 1.39±0.04 | 2.37±0.05 |
| N31 | 1.11±0.04 | 1.60±0.05 |
| N32 | 0.56±0.02 | 1.10±0.03 |
| N33 | 0.51±0.01 | 1.11±0.00 |
| N34 | 1.42±0.03 | 2.03±0.14 |
| N35 | 3.00±0.02 | 1.92±0.10 |
| N36 | 1.01±0.06 | 1.05±0.04 |
| N37 | 2.02±0.07 | 2.29±0.02 |
| N38 | 1.71±0.01 | 1.51±0.07 |
| N39 | 0.43±0.02 | 0.85±0.03 |
| N40 | 2.71±0.10 | 3.22±0.30 |
| N41 | 0.36±0.00 | 0.56±0.01 |
| N42 | 1.20±0.07 | 1.71±0.05 |
| N43 | 2.58±0.02 | 2.02±0.12 |
| N44 | 1.35±0.03 | 1.55±0.02 |
| N45 | 0.51±0.01 | 0.80±0.03 |
| N46 | 0.56±0.00 | 1.16±0.01 |
| N47 | 0.30±0.00 | 0.52±0.02 |
| N48 | 1.31±0.06 | 1.42±0.05 |
| N49 | 0.64±0.01 | 0.96±0.08 |
| N50 | 1.11±0.02 | 1.13±0.08 |
| N51 | 0.47±0.01 | 1.13±0.02 |
| N52 | 1.03±0.01 | 1.35±0.11 |
| N53 | 0.97±0.00 | 1.42±0.01 |
| N54 | 1.64±0.04 | 2.13±0.12 |
| N55 | 1.45±0.03 | 1.61±0.05 |
| N56 | 3.52±0.09 | 2.03±0.04 |
| N57 | 2.18±0.01 | 2.40±0.09 |
| N58 | 2.49±0.07 | 2.25±0.02 |
| N59 | 1.47±0.04 | 1.80±0.05 |
| N60 | 1.57±0.16 | 1.82±0.05 |
| N61 | 0.89±0.01 | 1.84±0.04 |
| N62 | 1.06±0.02 | 1.86±0.09 |
| N63 | 0.90±0.04 | 1.91±0.04 |
| N64 | 1.27±0.05 | 1.92±0.11 |
| N65 | 1.92±0.02 | 1.97±0.04 |
| N66 | 2.18±0.07 | 1.84±0.07 |
| N67 | 0.81±0.03 | 1.60±0.07 |
| N68 | 0.73±0.00 | 1.29±0.05 |
| N69 | 1.53±0.01 | 2.19±0.00 |
| N70 | 1.66±0.04 | 3.38±0.04 |
| C1 | 1.37±0.09 | 1.98±0.03 |
| C2 | 1.70±0.01 | 2.48±0.09 |
| C3 | 2.36±0.06 | 2.22±0.12 |
| C4 | 2.18±0.01 | 3.25±0.27 |
| C5 | 2.14±0.03 | 1.73±0.12 |
| C6 | 2.08±0.14 | 2.72±0.31 |
| C7 | 0.89±0.03 | 2.01±0.09 |
| C8 | 1.21±0.01 | 3.51±0.11 |
| G9 | 2.44±0.03 | 1.51±0.13 |
| C10 | 1.55±0.04 | 2.42±0.23 |
| C11 | 1.44±0.02 | 2.33±0.28 |
| C12 | 3.84±0.10 | 2.72±0.11 |
| C13 | 0.82±0.01 | 2.26±0.12 |
| C14 | 1.10±0.06 | 1.54±0.06 |
| C15 | 1.07±0.05 | 1.77±0.10 |
| C16 | 1.05±0.01 | 2.70±0.05 |
| C17 | 1.25±0.03 | 1.98±0.05 |
| C18 | 1.32±0.03 | 2.12±0.14 |
| C19 | 1.53±0.03 | 1.99±0.08 |
| C20 | 1.21±0.01 | 1.36±0.02 |
| C21 | 1.57±0.02 | 3.11±0.23 |
| C22 | 1.85±0.08 | 2.95±0.11 |
| C23 | 1.18±0.03 | 2.42±0.05 |
| C24 | 2.58±0.02 | 2.41±0.15 |
| C25 | 1.17±0.02 | 2.02±0.13 |
| C26 | 1.82±0.03 | 1.81±0.14 |
| C27 | 1.80±0.03 | 2.67±0.01 |
| C28 | 1.41±0.06 | 2.56±0.11 |
| C29 | 1.52±0.04 | 4.75±0.19 |
| C30 | 2.40±0.02 | 2.51±0.06 |
| C31 | 1.58±0.04 | 2.49±0.22 |
| C32 | 1.86±0.04 | 3.01±0.11 |
| C33 | 1.17±0.08 | 2.66±0.19 |
| C34 | 2.11±0.02 | 2.83±0.11 |
| C35 | 2.53±0.10 | 4.86±0.17 |
| C36 | 1.65±0.02 | 2.07±0.07 |
| C37 | 2.51±0.08 | 2.51±0.12 |
| C38 | 1.31±0.03 | 2.25±0.10 |
| C39 | 4.97±0.11 | 3.81±0.35 |
| C40 | 1.02±0.02 | 1.75±0.03 |
| C41 | 1.98±0.05 | 1.32±0.08 |
| C42 | 1.89±0.01 | 3.42±0.19 |
| C43 | 1.43±0.01 | 2.84±0.06 |
| C44 | 1.26±0.03 | 2.54±0.10 |
| C45 | 1.67±0.05 | 1.74±0.06 |
| C46 | 1.61±0.00 | 2.23±0.10 |
| C47 | 1.54±0.03 | 4.15±0.07 |
| C48 | 1.56±0.04 | 2.26±0.06 |
| C49 | 4.44±0.10 | 3.51±0.09 |
| C50 | 2.60±0.00 | 2.83±0.05 |
| C51 | 2.22±0.06 | 4.11±0.18 |
| C52 | 2.61±0.02 | 3.33±0.12 |
| C53 | 1.31±0.08 | 2.26±0.16 |
| C54 | 2.29±0.04 | 2.63±0.09 |
| C55 | 2.25±0.11 | 2.23±0.07 |
| C56 | 3.41±0.12 | 2.67±0.04 |
| C57 | 1.56±0.07 | 3.45±0.04 |
| C58 | 1.38±0.06 | 4.37±0.21 |
| C59 | 1.71±0.02 | 1.96±0.03 |
| C60 | 2.99±0.01 | 2.81±0.02 |

**Table S2.** The optimized MS conditions used for the analysis of 8-OHdG and 8-OHG.

| Compound | MRM  ion transition (*m/z*) | DP (V) | CE (V) | EP (V) | CXP (V) |
| --- | --- | --- | --- | --- | --- |
| 8-OHdG | 284.1>168.0 | 45.0 | 18.0 | 10.0 | 13.0 |
| [^15^N_5_]8-OHdG | 289.1>173.0 | 45.0 | 18.0 | 10.0 | 13.0 |
| 8-OHG | 300.1>168.0 | 60 | 10 | 22 | 13 |
| [^13^C^15^N_2_]8-OHG | 303.1>171.0 | 60 | 5 | 25 | 10 |

*DP* declustering potential, *CE* collision energy, *EP* entrance potential, *CXP* collision cell exit potential

**Figure S1.** Calibration curve of 8-OHdG and 8-OHG standard obtained in water and human urine for the evaluation of matrix effect.

**
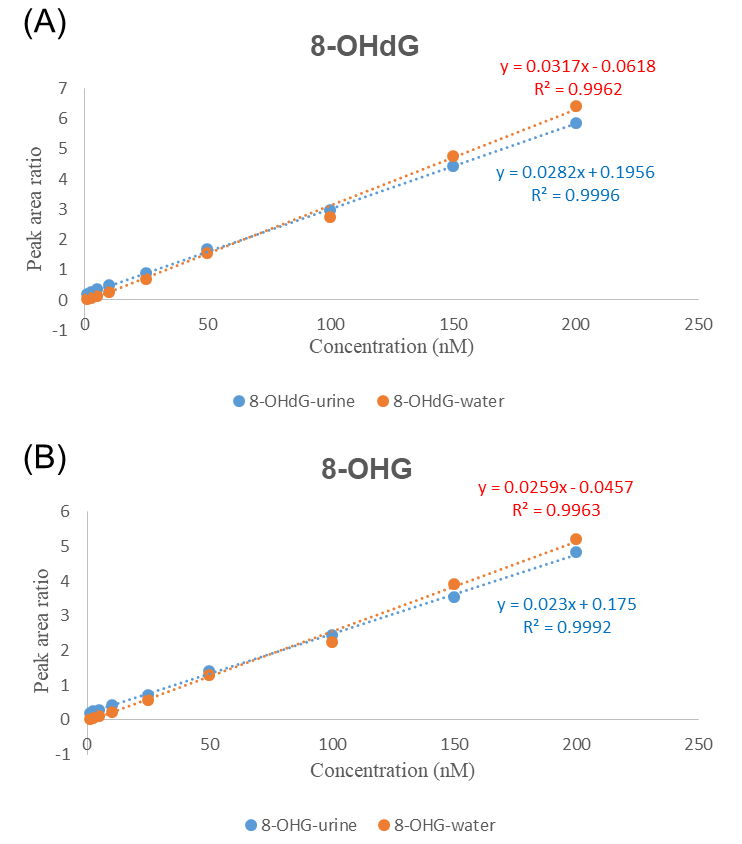
**
